# Conceptual Approaches to Modulating Antibody Effector Functions and Circulation Half-Life

**DOI:** 10.3389/fimmu.2019.01296

**Published:** 2019-06-07

**Authors:** Kevin O. Saunders

**Affiliations:** Laboratory of Protein Expression, Departments of Surgery, Molecular Genetics and Microbiology, and Immunology, Duke University Medical Center, Duke Human Vaccine Institute, Durham, NC, United States

**Keywords:** antibody engineering, Fc optimization, therapeutic antibodies, biologics, passive immunity, immunotherapy

## Abstract

Antibodies and Fc-fusion antibody-like proteins have become successful biologics developed for cancer treatment, passive immunity against infection, addiction, and autoimmune diseases. In general these biopharmaceuticals can be used for blocking protein:protein interactions, crosslinking host receptors to induce signaling, recruiting effector cells to targets, and fixing complement. With the vast capability of antibodies to affect infectious and genetic diseases much effort has been placed on improving and tailoring antibodies for specific functions. While antibody:antigen engagement is critical for an efficacious antibody biologic, equally as important are the hinge and constant domains of the heavy chain. It is the hinge and constant domains of the antibody that engage host receptors or complement protein to mediate a myriad of effector functions and regulate antibody circulation. Molecular and structural studies have provided insight into how the hinge and constant domains from antibodies across different species, isotypes, subclasses, and alleles are recognized by host cell receptors and complement protein C1q. The molecular details of these interactions have led to manipulation of the sequences and glycosylation of hinge and constant domains to enhance or reduce antibody effector functions and circulating half-life. This review will describe the concepts being applied to optimize the hinge and crystallizable fragment of antibodies, and it will detail how these interactions can be tuned up or down to mediate a biological function that confers a desired disease outcome.

## Introduction

Since the approval of the first monoclonal antibody by the FDA in 1986 (1), there has been a rapid increase in the number of available monoclonal antibodies or antibody derivatives. In 2015 there were 44 antibodies approved for human use in the United States and Europe (2). Consistent with an expected annual approval rate of six to nine additional antibodies (3), the number of approved antibodies and antibody-like biologics in the United States has climbed to more than 70 (1, 4). It is estimated that global sales of antibody-based products approach $60–75 billion in any given year (2–8). Therefore, many pharmaceutical companies are including antibody-like molecules in their development portfolio due to their high capacity to generate revenue.

Basic science continues to discover the underlying mechanisms of genetic disorders, cancer, and infectious diseases (9, 10). Elucidation of these mechanisms fuels the development of antibody-based biologics to counteract the abnormal biologic process that is causing disease. How the antibody counteracts the biologic process can be optimized for selectivity and potency by modifying the sequence of the antibody-based molecule to enhance or abrogate its interaction with the host immune system (11, 12). This concept is the foundation of antibody optimization efforts in industry laboratories as well as academic research laboratories. While most approved biologics are traditional antibodies, optimized antibodies like Orencia® (abatacept), Soliris® (eculizumab), Nplate® (romiplostim), and Removab® (catumaxomab) have paved the way for optimized antibodies as treatment options (11).

To optimize an antibody one must understand how the antibody is constructed and the role of each of its parts. An intact full-length antibody consists of two 50 kD heavy chains and two 25 kD light chains resulting in a 150 kD full-length, soluble immunoglobulin (13). Each heavy chain associates with a light chain through disulfide bonds and non-covalent interactions to form a heterodimer (14). The two heterodimers are paired together via disulfide bonds between the heavy chains (15, 16). Each heavy and light chain heterodimer includes the antigen binding fragment (Fab) composed of the light chain paired to the variable region of the heavy chain and the CH1 domain of the heavy chain constant region (17, 18). C-terminal to the Fab is the hinge, and the crystallizable fragment (Fc) (17, 18). The hinge region can be subdivided into upper, core, and lower hinge regions (19). The Fc includes the CH2 and CH3 domains of the heavy chain constant region (14).

**Figure 1:**
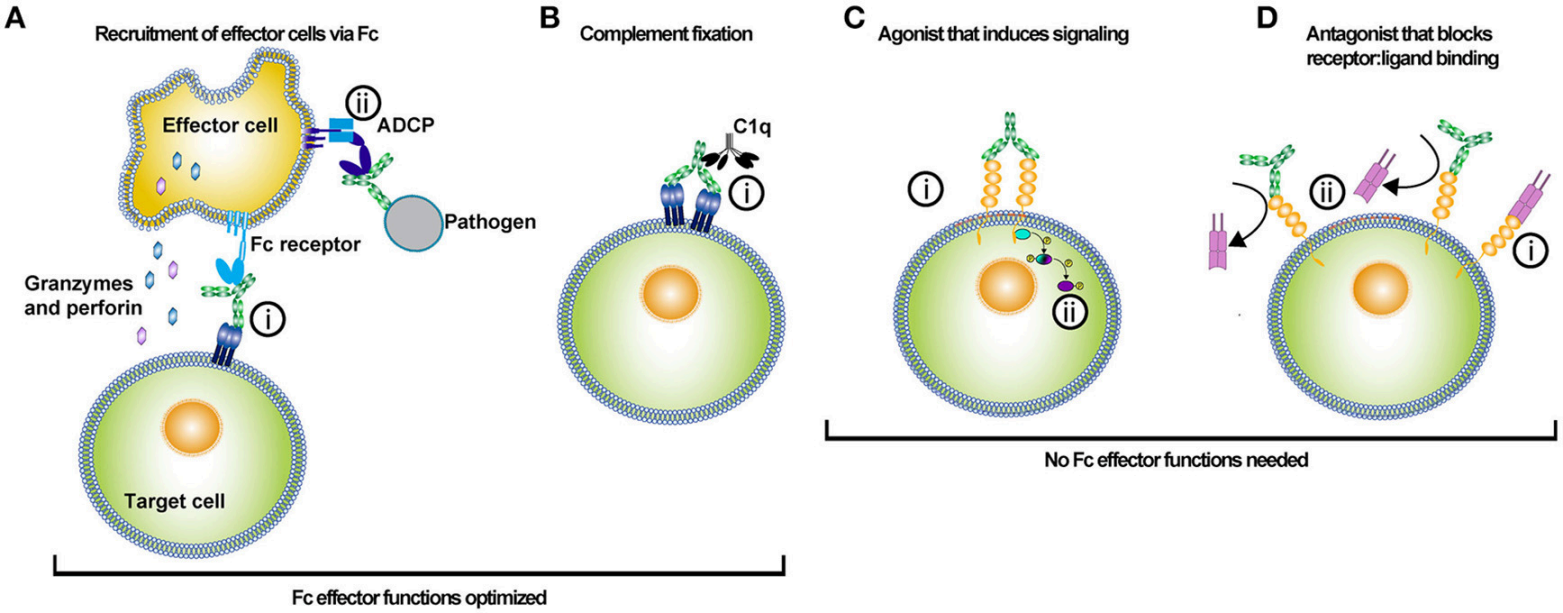
Uses of antibody-based biologics. The type of Fc optimization undertaken for an antibody depends on the desired function of the antibody *in vivo*. Antibody-based biologics are principally used as adaptor molecules for effector cell recruitment to targets, inducers of the classical complement pathway, agonists, or antagonists. **(A)** Antibody biologics can be used to opsonize target cells or pathogens and recruit effector cells to kill or phagocytose the target cell or pathogen. (i) Antibodies bind to the antigen via their Fabs and recruit effector cells via their Fc interacting with Fc receptors. Recruitment of the effector cell to the target cell and release of cytotoxic molecules, such as granzyme and perforin results in target cell killing. Alternatively, (ii) effector cells can bind to the Fc of an antibody that has opsonized a pathogen, and subsequently phagocytose the pathogen via antibody-dependent cellular phagocytosis (ADCP). Much of the Fc engineering effort has been focused on improving Fc affinity for various activating FcγRs. The improvement of Fc affinity for FcγRs has led to augmented ADCC and ADCP activity for the optimized antibody-based biologic. **(B)** Antibodies can kill target cells through the initiation of the complement pathway. The initial step in initiating the complement pathway is the binding of complement protein 1q (C1q). (i) C1q binds to the Fc of antibodies in complex with antigen. Specific mutations have been introduced into the Fc of antibodies to enhance Fc binding affinity to C1q. **(C)** Agonistic antibody-based molecules are designed to bind to ligand and induce signaling by crosslinking a membrane-bound receptor. (i) Binding of both Fab arms crosslinks the cell receptor and (ii) subsequent phosphorylation of intracellular signal transducers potentiates the receptor signaling. These types of antibody-based biologics do not require Fc function; hence these molecules are prime candidates for Fc optimization that silences FcγR and complement binding. **(D)** Antagonistic antibody-based biologics bind to a target molecule and prevent the function of that protein either directly or by blocking its ligand from binding. (i) Typically the ligand binds to its receptor, (ii) but in the presence of the antagonistic antibody the receptor binding site is competitively or allosterically blocked. The lack of ligand binding prevents intracellular signaling by the membrane-bound receptor. In this instance, Fc effector functions are not needed and thus mutations that silence Fc effector function can be employed to optimize these types of biologics.

**Figure 2:**
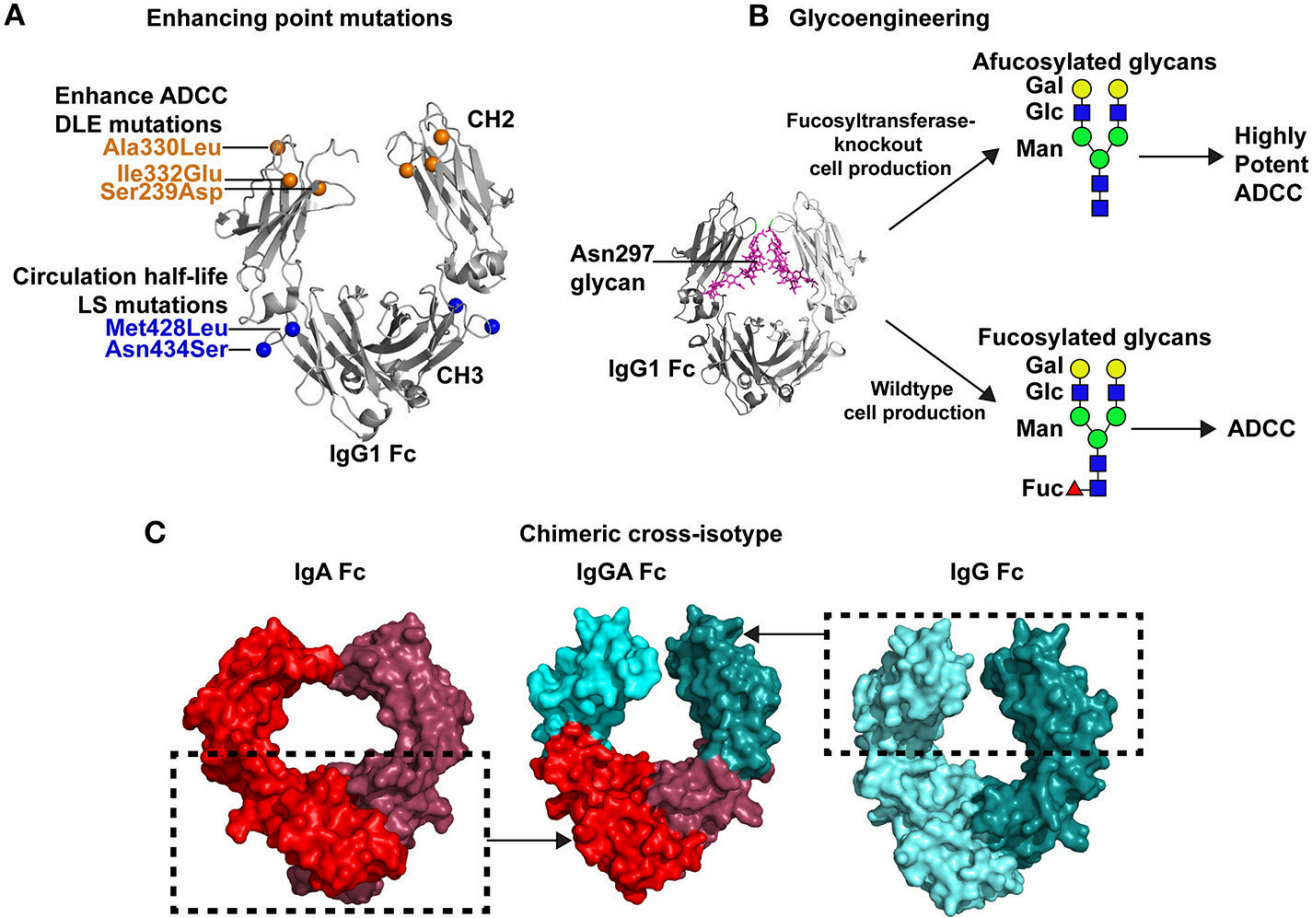
Strategies for improving antibody Fc-mediated effector functions. **(A)** Multiple point mutations have been identified that improve binding affinity of Fc for specific FcγRs. In some instances a single FcγR, such as FcγRIIIa is the receptor of interest. Directed evolution, alanine scanning, or structure-guided design have been used to identify these mutations. An example of these mutations is the DLE (Ser239Asp/Ile332Glu/Ala330Leu) set of mutations that are shown in the crystal structure of the Fc by orange spheres (PDB:3DO3; 42). These mutations improve ADCC activity. Additionally, mutations can be inserted that improve antibody circulation *in vivo*. The LS mutations, depicted by blue spheres, (Met428Leu/Asn434Ser) are one example of antibody half-life extension mutations. **(B)** Antibody effector functions can be enhanced by glycoengineering the Fc domain. The Fc domain contains a N-linked glycan at position 297. A crystal structure of the IgG1 Fc (gray) and the N297 glycan (magenta) are shown (PDB:4BYH). Expression of antibody in wildtype cells results in a fucosylated complex glycans present at N297. However, specialized cells have been created with fucosyltransferase knocked out, which results in afucoylated glycans at Asn297. Antibodies with afucosylated glycans exhibit upto 50-fold more potent ADCC than the same antibody with a fucosylated glycan at Asn297 (60). Green circles, mannose; blue squares, GlcNAc2; yellow circles, galactose; and red triangles, fucose. **(C)** Antibody effector functions can be improved by expanding the breadth of Fc receptors capable of interacting with Fc. To improve antibody effector function the Fc of a single antibody can be engineered to bind to Fc receptors for multiple antibody isotypes. This concept has led to the design of cross-isotype IgGA antibodies (center) where the IgG1 CH2 a1 loop residues 245–258 and the IgG1 CH3 domain (cyan) were exchanged with the structurally analogous regions of IgA (54). The regions inside the dashed box were combined to create a chimeric cross-isotype Fc. The IgG1 segments are colored light and dark cyan (right, PDB: 3DO3), and the IgA segments are colored light and dark red (left, PDB: 1OW0). The cross-isotype Fc is capable of binding to FcγRI and FcαRI, hence either of these Fc receptors can be used to recruit diverse effector cells to target cells (54).

The constant region of antibodies also contributes to the sequence variation of the heavy chain. The variable region of the heavy chain recombines with the heavy chain constant region to produce a full-length heavy chain (20, 21). The antibody can vary in isotype depending on whether the alpha, mu, gamma, epsilon, or delta constant region gene segment is recombined with the variable region (22). Among the human gamma gene segments there are 4 different subclasses designated as gamma 1, 2, 3, and 4, which are approximately 90% identical to each other (1). In a clinical context, each subclass is important since each subclass specializes in the elimination of different types of pathogens (23). For example, there is an association between deficiency in IgG2 antibodies and infection with encapsulated bacteria (24). The molecular basis of the association may be a diminished antibody response to polysaccharide antigens in individuals lacking IgG2 antibodies (25). For antibody engineering, the different isotypes and subclasses are important for antibody optimization since the sequence variation occurs at sites that determine affinities and specificities for FcRn, Fc alpha receptor, Fc gamma receptors, and complement protein C1q (26). There are 5 Fc gamma receptors (FcγR) that activate effector cells upon binding to IgG. Among the activating receptors there are FcγRI, FcγRIIa, FcγRIIc, FcγRIIIa, and FcγRIIIb (27). There is one inhibitory Fc gamma receptor—FcγRIIb (28, 29). The FcγRs are polymorphic, where certain alleles exhibit higher affinity for Fc than others. For example Val158 allelic variants of FcγRIIIa bind with higher affinity to IgG1 Fc than the Phe158 allelic variant (30). Antibody binding to these receptors can facilitate the recruitment of effector cells to opsonized target cells or opsonized pathogens for clearance [Figure 1A; (2)]. Therefore, changes to the sequence and post-translational modification of the Fc and hinge regions of antibodies allows one to manipulate the effector functions and circulation of a given antibody or antibody-like protein (31). In addition to sequence variation, the Fc region also contains an N-linked glycosylation site at residue 297, which is important for Fc structure and function (32). Most clinically approved antibody-based products are of the gamma isotype, subclass 1 (IgG1) (2). There are currently 3 IgG2 antibodies that are approved for use in the United States (2). IgG3 has long hinge region prone to proteolytic cleavage (23), and exhibits a reduced half-life relative to other IgG subclasses (33). For these reasons it has it has not been the subclass of choice for biologics. Since most clinically-approved antibodies are of the gamma isotype (2), the optimization of antibody binding to FcγRs has been the major focus of the Fc engineering field. However, it is important to note that IgA, IgM, and IgE isotypes have Fc receptors as well, which can be exploited by Fc engineering as discussed below (34–36).

In this review, the approaches utilized to optimize or eliminate Fc interactions with host proteins will be discussed. This review will focus on changes to Fc sequence and glycosylation as a means to modulate Fc function. While Fc optimization is presented as two distinct categories of either enhancement or abrogation of Fc binding the review will describe how a single mutation can have both effects; thus, the two categories are not mutually exclusive. Ultimately, the reader will gain knowledge of how to alter the Fc region of an antibody to change its immunologic properties.

## Antibody Fc Mutations for the Improvement of Effector Functions

### Enhanced FcγR Binding

Engagement of FcγRs is required for antibody effector functions, such as antibody-dependent cellular cytotoxicity (ADCC) and antibody-dependent cellular phagocytosis (ADCP) (37). Below, modifications that affect binding to FcγRs which result in enhanced ADCC and ADCP are described.

#### Point Mutations to Enhance FcγR Binding

The Fc of antibodies has been optimized using multiple approaches in attempts to increase binding affinity to selected FcγR (Table 1 and Figure 2). Guided by the 3.2 Å structure of the Fc of IgG1 (61), Shields et al. performed alanine scanning mutagenesis of the solvent exposed amino acid residues on Fc (38). Antibodies encoding Fc regions with alanine mutations were screened for their binding to FcγRI, FcγRIIa, FcγRIIb, FcγRIIIa, and FcRn. Each individual mutation was subdivided into improved or reduced binding to each FcγR and FcRn. Twenty-seven individual mutations increased binding to at least one FcγR or FcRn. In an attempt to engineer an Fc that bound strongly to FcγRIII—a receptor that mediates ADCC (62, 63)—alanine mutations at different sites were combined into one modified Fc. The combination of Ser298Ala, Glu333Ala, and Lys334Ala mutations (sometimes referred to as the AAA mutations) had an additive improvement on the affinity of IgG1 for FcγRIIIa (Table 1) (38). The improved binding to FcγRIIIa translated to 50–100-fold more potent killing *in vitro* of Her2+ cells by the antibody Herceptin when Ser298Ala, Glu333Ala, and Lys334Ala mutations were incorporated.

**Table 1:** Fc modifications to enhance antibody effector function.

**Modifications or mutations (reference)**	**Abbreviated name**	**Phenotype**	**Enhanced effector function**
Ser298Ala/Glu333Ala/Lys334Ala (38)	AAA	• Enhanced FcγRIIIa affinity	ADCC
Ser239Asp/Ala330Leu/Ile332Glu (39, 40)	DLE	• Increased FcγRIIIa affinity • Low binding to inhibitory FcγRIIb	ADCC ADCP
Ser239Asp/Ile332Glu (39, 40)	DE	• Increased FcγRIIIa • Strong binding to inhibitory FcγRIIb	ADCC ADCP
Gly236Ala/Ser239Asp/Ala330Leu/Ile332Glu (41–43)	GASDALIE	• Increased binding affinity to FcγRIIa and FcγRIIIa • Only a small increase to FcγRIIb	ADCC
Gly236Ala (40)	GA	• Increases FcγRIIa affinity • No change in FcγRIIb affinity • Decreased FcγR1	ADCP
Ser239Asp/Ile332Glu/Gly236Ala (40)	DAE	• Recovers FcγRI binding lost by Gly236Ala • Increases FcγRIIIa and FcγRIIa • Enhanced FcγRIIb binding	ADCC ADCP
Leu234Tyr/Gly236Trp/Ser298Ala (44)	YWA	• Improved FcγRIIIa affinity when present in 1 heavy chain constant region • Used in asymmetric Fc design with DLE	ADCC
Phe243Leu, Arg292Pro, Tyr300Leu, Val305Ile, and Pro396Leu (45)	Variant 18	• Enhanced FcγRIIa and FcγRIIIa off-rates • Less than 2 fold enhancement of FcγRIIb	ADCC
Lys326Trp/Glu333Ser (46)		• Increased C1q binding • CDC activity was comparable to Lys326Trp, but improved versus wildtype Fc • Decreased ADCC activity	CDC
Lys326Ala/Glu333Ala (46)		• Increased C1q binding • Preserved ADCC activity	CDC
Lys326Met/Glu333Ser (46)		• Increased CDC activity • Preserved ADCC activity	CDC
Cys221Asp/Asp222Cys (47)		• Increased C1q binding • Preserves FcγRIII affinity and ADCC	CDC
Ser267Glu, His268Phe, and Ser324Thr (48)	EFT	• Increased C1q binding • Ser267Glu increased inhibitory FcγRIIb affinity • Decreased ADCC/ADCP	CDC
His268Phe and Ser324Thr (48)	FT	• Improved CDC • Functions with ADCC and ADCP enhancing mutations • Less potent CDC than EFT	CDC
Glu345Arg (49)	Arg345	• Increased C1q binding • IgG1 hexamer formation	CDC
IgG1/IgG3 cross-subclass (50)	1133 1131	• Increased C1q binding • Preserves ADCC activity	CDC
IgG2/IgG3 cross-subclass (51)	IgG 3-3-3/2-3 IgG 2-2-3-2	• Increases C1q and C4b binding	CDC
4-domain cross-isotype (52)	γ*γγα*	• Decreased FcγRI binding • Decreased Polymeric Ig receptor binding • Decreased half-life	CDC
Tandem cross-isotype (53)	IgG1/IgA2	• Bound to FcγRs, FcαRI, and FcRn • Decreased C1q binding	ADCC
Chimeric cross-isotype (54)	IgGA	• Bound to FcγRI, FcγRIIa, FcαRI • Lost FcRn	ADCC ADCP CDC
Multimeric IgG (55)		• Increased C1q • Increased FcγRI and FcγRIII	CDC
Galactosylation (56, 57)		• Increased C1q	CDC
Biantennary glycan at N297 (58, 59)		• Improved binding to FcγRIIIa	ADCC
Afucosylated glycan at N297 (60)		• Increased binding to FcγRIIIa	ADCC

In a directed evolution approach, Lazar et al. used a computational algorithm to calculate amino acid substitutions that would be predicted to improve the interaction between Fc and FcγRIIIa (39). They also generated a set of quality improvement mutations that would be predicted to improve stability and solubility. Ser239Asp and Ile332Glu in the CH2 domain individually improved FcγRIIIa binding affinity by one log compared to the wildtype Fc (Figure 2A). To maximize binding these two mutations were combined into one Fc construct which resulted in an approximately 2-log enhancement in binding affinity for FcγRIIIa compared to the wildtype Fc. However, an unwanted increase in binding to the inhibitory FcγRIIb was also conferred by the Ser239Asp/Ile332Glu double mutant (39). This undesired effect was partially negated by adding an Ala330Leu mutation to the Ser239Asp/Ile332Glu variant (Figure 2A). The triple mutant Ser239Asp/Ile332Glu/Ala330Leu (commonly referred to as DLE) was introduced into the Fc of anti-cancer antibody Trastuzumab. Compared to wildtype Trastuzumab, Trastuzumab with the DLE mutations had 2-log more potent ADCC killing of Her2+ cancer cell lines expressing low or high levels of cancer antigen (39). The DLE mutations also increased ADCC activity of an anti-integrin antibody, MEDI-522, against a human melanoma cell line (64). Similarly, these mutations increased ADCP by the anti-CD20 cancer antibody Rituximab (39). At the cellular level the DLE mutations function to recruit higher numbers of natural killer cells per target cell coated with the DLE-optimized antibody compared to wildtype antibody (65). Approximately 90% of the recruited NK cells kill cell the target upon first contact, and then move to a second cell for killing (65). Thus, single NK cells kill more target cells per contact and more target cells over time (65). This increase in recruitment and more efficient killing conferred by the DLE-optimized antibody was shown to be due to enhanced FcγRIII-mediated signaling as measured by ZAP70 phosphorylation (65).

The crystal structure of the Fc containing the Ser239Asp/Ile332Glu/Ala330Leu mutations was solved to understand how these mutations affect FcγRIIIa binding. The structure showed an open conformation of the Fc where the two CH2 domains were separated from each other by an additional 30 Å compared to wildtype Fc (66). Thermostability measurements suggested that the opening of the CH2 domain could be because the CH2 domain was less stable or more flexible upon addition of the Ser239Asp/Ile332Glu/Ala330Leu mutations (66). The structure of the optimized Fc was modeled interacting with FcγRIIIa, to determine whether the Ser239Asp/Ile332Glu/Ala330Leu mutations created additional interactions with the FcγRIIIa. Indeed, the structural model suggested additional hydrogen bonds between Ser239Asp/Ile332Glu in the Fc and Lys158 in the FcγRIIIa. Ala330Leu potentially created more hydrogen bonds with Ile85 in the FcγRIIIa as well (66). Additional electrostatic and hydrophobic interactions were also suggested by the structural model (66).

Mimoto et al. engineered an asymmetric Fc that combined the DLE mutations with their newly-identified Fc optimization mutations (44). In a large saturating mutagenesis screen, they examined the binding of 1,000 single Fc mutants to identify mutations that improved FcγRIIIa binding when present in only one of the heavy chains within an IgG molecule. They selected three mutations, Leu234Tyr, Gly236Trp, and Ser298Ala (termed YWA mutations), from their screen to incorporate into one heavy chain constant region. Since the DLE mutations had been shown to increase FcγRIIIa binding they incorporated these mutations into the other heavy chain constant region. Antibodies bearing YWA mutations in one heavy chain and DLE in the other mediated ADCC of tumor antigen-expressing cells *in vitro* more potently than symmetrical antibodies that contained only the YWA or DLE mutations (44). Thus, this asymmetric Fc design enables one to incorporate multiple optimization mutations to additively improve Fc function.

Macrophages utilize FcγRIIa to phagocytose antibody-opsonized antigens (67). To increase Fc receptor binding to FcγRIIa, Richards et al. screened 900 Fc variants for binding to FcγRIIa and identified Gly236Ala substitution alone increased the binding affinity approximately 6-fold for both His131 and Arg131 alleles of FcγRIIa (40). Unfortunately, the addition of Gly236Ala into IgG1 Fc reduced the IgG1 affinity for the activating receptor FcγRI (40). To recover the FcγRI binding, previously reported Ser239Asp/Ile332Glu mutations were introduced into the IgG1 Fc. This triple combination of mutations showed a 3-fold increase in FcγRI up to 70-fold increase in affinity for FcγRIIa, and up to a 31-fold increase in affinity for FcγRIIIa. The Ser239Asp/Ile332Glu/Gly236Ala mutations enhanced *in vitro* FcγRIIa-dependent phagocytosis and FcγRIII-dependent ADCC activity of an IgG1 targeting adenocarcinoma cell lines (40).

The activating receptor FcγRIIa is 90% similar to the inhibitory receptor FcγRIIb (68), and thus the increase in FcγRI and FcγRIIIa affinity for Ser239Asp/Ile332Glu/Gly236Ala was accompanied by a 13-fold enhancement in binding to FcγRIIb. To compare the binding of both the activating and inhibitory FcγRIIs the ratio of binding was determined. The ratio of binding between the activating FcγRIIa and inhibitory FcγRIIb receptors was higher for Gly236Ala and the Ser239Asp/Ile332Glu/Gly236Ala than wildtype IgG1 (40). Thus, the ratio may be most important for determining the final functional activity of antibodies encoding the Ser239Asp/Ile332Glu/Gly236Ala mutations. Smith et al. attempted to improve the binding ratio of FcγRIIa to FcγRIIb by combining related sets of mutations to generate Gly236Ala/Ser239Asp/Ala330Leu/Ile332Glu (referred to as GASDALIE) (41). This collection of mutations increased binding affinity to FcγRIIIa encoding the low affinity allele Phe158 by 30-fold, most likely because of increased electrostatic interactions between the Fc and FcγRIIIa (41, 42). Similarly, binding affinity to FcγRIIa was improved 25-fold (41). FcγRIIb binding affinity affinities were only slightly increased, which resulted in a FcγRIIa to FcγRIIb affinity ratio of 11.6 compared to 1.6 for wildtype IgG1 (41). In a second experiment, investigators attempted to optimize Fc while avoiding any mutations that increased FcγRIIb binding. Using yeast display the investigators identified mutations that increased FcγRIIIa binding and reduced FcγRIIb binding. Upon making their mutant libraries and expressing them on the surface of yeast they used bead depletion to remove antibody Fc variants that bound to FcγRIIb. After FcγRIIb-bead depletion, the library of remaining Fc regions was screened for binding to recombinant FcγRIIIa. Using two different libraries, seven single mutations appeared to lack FcγRIIb binding while improving FcγRIIIa binding. These mutations were introduced into the Fc region of IgG1 individually as well as in various combinations. Combinations of Phe243Leu, Arg292Pro, Tyr300Leu, Val305Ile, and Pro396Leu mutations slowed the off-rates of Fc binding to FcγRIIa and FcγRIIIa relative to wildtype Fc without increasing binding to the inhibitory FcγRIIb receptor. The Fc that included all five mutations—termed variant 18—had a 10-fold improvement in affinity for FcγRIIa and FcγRIIIa, and <2-fold increase in FcγRIIb affinity. The variant 18 Fc exhibited potent ADCC activity against colon, ovarian, and breast cancer cell lines *in vitro* for several different antibodies (45). *In vivo*, an IgG1 encoding Phe243Leu, Arg292Pro, Tyr300Leu, Val305Ile, and Pro396Leu mutations conferred a significant increase in survival in a lethal ovarian tumor transplantation model (45). These mutations have shown promise in treatment of cancer in humans as well. Anti-HER2 monoclonal antibody Margetuximab, which contains Phe243Leu/Arg292Pro/Tyr300Leu/Val305Ile/Pro396Leu optimization mutations, exhibits improved ADCC activity compared to the standard of care antibody trastuzumab (69). In one clinical trial 78% of the response-evaluatable patients who received Margetuximab showed a reduction in tumor size (70), highlighting the potential promise for Fc optimization to improve disease treatment.

#### Glycoengineering to Enhance FcγR Binding

The Fc of IgG1 contains a single N-linked glycosylation site at position 297. The glycan present at N297 typically consists of two N-acetylglucosamine (GlcNAc), three mannose, and two more GlcNAc linked to the mannose to form a biantennary complex glycan (71). The two GlcNAc are linked to mannose through either a β1,2 linkage to α-3 or α-6 of the mannose. Thus, each arm of the glycan can be distinguished as the α1,3 or α1,6 arm depending upon how the mannose and GlcNAc_2_ are linked (71). Additional fucose, galactose, sialic acid, and GlcNAc can be added to the core glycan structure (Figure 2B). IgG found circulating in human sera are generally fucosylated, however during recombinant IgG production the glycan composition can be altered by expressing the antibody in plant cells, knocking in or knocking out specific glycosidases, or *in vitro* enzymatic digestion of the glycosylated IgG (Figure 2B) (72). Since both heavy chains are glycosylated it is possible for a single IgG molecule to have significant glycan heterogeneity (71). The glycan has direct effects on FcγR binding. The Asn297 glycan on the Fc can clash with glycans on the FcγRIII protein, which results in poor engagement of effectors cells that mediate ADCC. Also, nuclear magnetic resonance studies have shown that Fc regions containing different glycans at Asn297 adopt different hinge region conformations (73). Since the hinge region is contacted by FcγRs, the glycosylation of N297 indirectly affects the ability of the Fc to interact with FcγRs. Thus, optimization of Fc glycosylation has been important for producing antibody biologics with a desired function.

To modulate antibody activity several studies have modified antibody Fc glycosylation by expressing or inhibiting enzymes in the producer cells. Expression of β(1,4)-N-acetylglucosaminyltransferase III when expressing IgG gives an antibody glycosylated at N297 that has a biantennary glycan and has better ADCC activity (58). Davies et al. produced anti-CD20 IgG1 under these conditions and found the antibody had 10–20-fold more potent FcγRIIIa-dependent killing of CD20+ cells (59). Despite these results the importance of bisecting GlcNAc is debated, and the removal of fucose has been asserted as an alternative hypothesis [Figure 2B; (74)]. Antibodies deficient in fucose have been shown to have 50-fold higher binding to FcγRIIIa and enhanced ADCC activity (60). The enhancement of afucosylated antibody binding to FcγRIIIa is higher for the high affinity Val158 allele compared to the Phe158 allele, but both alleles show an increase in binding to afucosylated IgG1 compared to fucosylated IgG1 (40, 75). The most dramatic increase in binding by afucosylated Fc is for glycosylated FcγRIIIa (76), with the removal of the Asn162 glycan in FcγRIIIa completely abrogating this enhanced binding (76). The mechanism for glycosylated FcγRIII recognition was later determined by structural studies, which showed that the afucosylated Asn297 glycan interacts with the Asn162 glycan on FcγRIII (77). The addition of fucose to the Fc glycan creates clashes with the GlcNAc_2_ on FcγRIIIa, providing a structural explanation for why afucosylated antibodies bind better to FcγRIIIa (77). The approval of mogamulizumab (POTELIGEO®) in Japan marked the first approval for human use of an afucosylated antibody with enhanced ADCC activity (78–80).

#### Enhancing FcγR Binding by Exchange of Fc Domains Across Isotypes (Cross-Isotype Antibodies)

In addition to increasing affinity for receptors by introducing point mutations or modifying glycans, the Fc can be optimized to engage a wider range of Fc receptors (Figure 2C). As stated above, Fc receptors for isotypes other than gamma exist on particular leukocytes. By creating a Fc region that can interact with multiple Fc receptors, such as FcγR and FcαRI, one creates an antibody with expanded, novel abilities to engage effector cells (2). Neutrophils are the most abundant leukocyte in the body, and they engage Fc of IgA antibodies via the FcαRI (81, 82). Single domains of IgA2 were appended to end of the gamma 1 constant region creating a four-domain constant region (CH1g-CH2g-CH3g-CH3a) (52) in an attempt to engage FcγRs and FcαRI. To make the constant region more similar to the alpha constant region the CH1 domain of gamma 1 was substituted for the alpha 1 constant region domain (CH1a-CH2g-CH3g-CH3a). These four-domain cross-isotype IgGA chimeric antibodies bound to J chain similar to natural IgA2 but had reduced transport by polymeric Ig receptor. The four-domain, cross-isotype antibodies also had a 3–5-fold decrease in FcγRI affinity, and possessed the short serum half-life of IgA2 instead of the protracted serum circulation of IgG1. Despite these shortcomings, the four-domain cross-isotype IgGA design was capable of mediating complement-dependent lysis of sheep red blood cells and appeared to be more pH-resistant than IgG1 (52). In a similar approach a second type of cross-isotype Fc has been created by fusing the gamma 1 and alpha constant regions together to create a tandem G1-A Fc region (53). In this design the hinge, CH2, and CH3 of IgA2 is fused to the C-terminus of IgG1. This tandem cross-isotype IgG/IgA design had similar expression levels, antigen binding, and thermostability as antibodies made in the IgG1 format. *In vitro*, the tandem cross-isotype IgG/IgA bound to FcαRI and FcγRI, FcγRII, FcγRIIIa, and FcRn with affinities similar to wildtype IgA and IgG, respectively. This binding to various FcRs translated to the antibody mediating ADCC activity with polymorphonuclear cells and NK cells. C1q binding to the tandem cross-isotype IgG/IgA was reduced 3-fold compared to the IgG1 format of the antibody. Lastly, in BALB/c mice, tandem IgG/IgA circulated with a half-life similar to that of IgG1. In a third design, Kelton et al. created a cross-isotype antibody by exchanging the CH3 domain and CH2 α1 loop residues 245–258 (PKPKDTLMISRTPE) of the gamma 1 constant region with that of the alpha constant region [Figure 2C; (54)]. The chimeric Fc possessed the ability to bind to FcγRI, FcγRIIa, and FcαRI (54). Antibodies made in this IgGA format were capable of mediating ADCC with polymorphonuclear cells, mediating ADCP with macrophages, and activating complement (54). However, this design lacked binding to neonatal Fc receptor which regulates antibody half-life (54). Thus, further optimization would be required for effective *in vivo* use of this design. In total, these designs show the promise of the concept of engaging a wide range of effector cells for antibody Fc optimization.

#### IgG Multimerization Augments FcγR Binding

Multimerizing IgG has shown promise in the treatment of autoimmune diseases (83, 84). The IgG multimers are constructed in various ways including the addition of heterologous multimerization domains such as isoleucine zippers (83), another hinge region at the N-terminus of the natural hinge, or another hinge region at the C-terminus of the CH3 domain (83). Similarly, hexamers of IgG have been created by appending the IgM tailpiece to the C-terminus of the IgG1 Fc and creating a cysteine bond at position 309 (85, 86). The multimeric IgG formed by the addition of the IgM tailpiece bound strongly to FcγRI, FcγRIIa, and FcγRIIIa and bound weakly to FcγRIIb and FcγRIIIb (85, 86). Across the various designs, multimeric IgG bound to a higher magnitude than monomeric IgG to FcγRI, FcγRIIb, and FcγRIII (83, 85). Such molecules have shown promise in preclinical models of arthritis, neuropathy, and autoimmune myasthenia gravis (83, 84, 87). Hence the multimeric IgG platform is being further optimized to fine-tune the immune receptors, such as FcRn that can interact with the multimer (55).

### Enhancing Complement Fixation

#### Point Mutations to Increase C1q Binding and Complement-Dependent Cytotoxicity (CDC)

Antibodies can exert cytotoxic effects by engaging the complement pathway. The initial step of this process is the binding of C1q to the CH2 domain of an antibody-opsonized antigen [Figure 1B; (88)]. Alanine scanning mutagenesis of the human IgG1 Fc identified Asp270, Lys322, Pro329, and Pro331 as essential for C1q binding to the Fc (89), although Fc from different species utilize different residues for binding to C1q (90). To increase Fc binding to C1q Idusogie et al. identified Lys326 and Glu333 as proximal to the core binding site of C1q within the Fc. The importance of these residues was first tested by alanine mutagenesis. Introducing Lys326Ala and Glu333Ala increased C1q binding and CDC activity by 50%. To optimize the Fc for binding to C1q various amino acids were introduced at 326 and 333 individually and in combination. The combination of Lys326Trp and Glu333Ser increased C1q binding by 5-fold (46). However, CDC activity conferred by the Lys326Trp/Glu333Ser double mutant Fc was the same as the Lys326Trp single mutant, and the Fc lost the ability to mediate ADCC. In instances when ADCC activity is also important, mutating positions 326 and 333 to two alanines, or mutating positions 326 and 333 to methionine and serine, respectively, provided an increase in CDC without hindering ADCC (46). Similar mutagenesis experiments have been done for the hinge region to determine whether it affects C1q binding and CDC activity. The mutation of the hinge region was not intuitive since C1q binds to the CH2 domain below the hinge region. However, it was shown that in the upper hinge region substituting Trp in various combinations at positions 222, 223, 224, and 225 increased C1q binding and increased CDC activity relative to wildtype IgG1 (47). ADCC activity and FcγRIIIa binding were unchanged by these modifications (47). Cys221Asp and Asp222Cys alone or in combination with Trp substitutions also increased C1q and CDC activity (47). Thus, the hinge of human IgG1 modulated C1q binding to the CH2 domain (47).

To identify other point mutations that improve CDC activity of IgG1, Moore et al. made 38 Fc variants of an anti-CD20 antibody and screened them *in vitro* for their ability to mediate CDC against Raji cells (48). Among the 38 variants, three variants encoding Ser267Glu, His268Phe, and Ser324Thr (termed the EFT mutations) changes were identified as having more potent CDC activity vs. wildtype IgG1 (48). The largest improvement in CDC activity was achieved when the three mutations were combined into one Fc variant (48). Correlation analyses suggested the improvement in CDC potency was due to increased C1q binding (48). The triple EFT mutations had increased binding to the inhibitory FcγRIIb, which presumably limited its ADCC and ADCP activity. The addition of ADCC and ADCP-enhancing mutations to the EFT mutations restored ADCC and ADCP function back to wildtype IgG1 levels, but did not confer an improvement (48). The increased binding to FcγRIIb could be reduced by eliminating the Ser267Glu from the EFT mutations, however this change came a cost of reduced CDC potency. The His268Phe and Ser324Thr mutations were then capable of being combined with ADCC and ADCP-enhancing mutations to create a single Fc with improved CDC, ADCC, and ADCP activity (48). This study highlights the complicated balance between optimizing one effector function without decreasing another effector function.

#### Insertions and Deletions to Increase to Increase C1q Binding and Complement-Dependent Cytotoxicity (CDC)

Hinge length is important for C1q recognition of antibodies or antibody-based proteins. IgG3 has a distinct extended hinge of 62 amino acids that arises from the duplication of 3 exons that encode for part of the core hinge region (91). For IgG3 antibodies complement activation is increased by shortening its hinge region (92). While complete removal of the hinge ablates CDC function, a hinge of 15 amino acids instead of 62 amino acids exhibited 10-fold more potent CDC activity (92). More specifically, removal of the three repeats regions within the core hinge does not eliminate CDC, but instead improved CDC potency for anti-bacterial antibodies (93). This result for IgG3 is in contrast to IgG1 where two amino acid deletions in the core hinge region reduced C1q binding, CDC activity, and ADCC (47).

#### Cross-Subtype Antibodies to Improve C1q Binding

IgG1 is the preferred subclass for antibody biopharmaceuticals over IgG3 since the long hinge of IgG3 complicates large scale production of the antibody (2). However, IgG3 possesses the best *in vitro* binding to C1q (94). As a means to enhance IgG1 C1q binding, domains of IgG3 where substituted for IgG1 domains to create IgG1/G3 cross-subtype antibodies (50). These chimeras eliminated the difficulty of purifying antibodies with long hinge regions but capitalized on IgG3 effector functions. In one of the best variants, termed 1133, the CH1 and hinge region from IgG1 was fused to the Fc from IgG3 (50). Its ADCC activity and antigen binding were unchanged, while, its CDC activity and C1q binding were enhanced relative to wildtype IgG1 or IgG3 (50). Furthermore, the 1133 design allowed for CDC activity when the antigen levels were low (95). However, the 1133 Fc variant lacked protein A binding, which is important for easy purification of the antibody. Thus, an antibody with the CH1, hinge, and CH3 of IgG1 and CH2 of IgG3 was constructed since C1q binds the CH2 domain and protein A binds the CH3 domain. This variant had improved CDC activity and the ability to bind protein A (50). The molecular basis for the improved binding of the chimeras is presumed to be the amino acid differences in the CH2 domain that are proximal to the C1q binding site in the tertiary structure of the Fc. In mutagenesis experiments aiming to define the amino acids required for C1q binding to IgG1 and IgG3 K322 was found to be important for both subclasses, but dependence on other amino acids varied between subclasses. For example, P331 was required for CDC activity of IgG1 (89), but had only a modest effect on IgG3 CDC activity (96). These results indicate that C1q binding differs between IgG3 and IgG1 thus chimeric antibodies may be able to enhance binding by combining Fc:C1q interactions from both gamma subclasses. While the goal of Fc designs has been to boost IgG1 activity, cross-subclass designs have also been used to confer activity to functionally silent subclasses. IgG2 and IgG4 have very little ability to mediate CDC compared to IgG1 or IgG3 (51). However, replacing the CH2 domain of IgG2 with that of IgG3 can instill CDC activity to the otherwise IgG2 Fc (51). Similarly, IgG4 differs from IgG1 at position 331, which has been shown to be proximal to the C1q binding site (90). Changing the IgG4 residue at position 331 to match IgG1 conferred a moderate level of CDC. Therefore, if one knows the key residues for mediating an effector function they can be introduced into functionally silent Fc domains to confer specific functions.

#### IgG1 Hexamer Formation Boosts C1q Binding and CDC Activity

The multimerization of IgG by binding to antigen is known to enhance C1q binding substantially (97). To engender multimerization of the IgG in the absence of antigen, analysis of IgG structures identified position 345 as an amino acid that could facilitate multimerization between the Fc regions of different antibodies (49). The IgG structure suggested that the introduction of a positively charged amino acid would confer Fc:Fc interactions. Thus, a Glu345Arg mutation was introduced into the IgG1 Fc. Electron microscopy and mass spectrometry confirmed that this mutation resulted in monomeric IgG1 as well as multimeric IgG1 linked via the Fc (49). The multimeric IgG possessed higher binding to C1q and more potent lysis of a Burkitt's lymphoma cell line (49). Interestingly, the Glu345Arg mutation not only increased CDC activity by IgG1, but also IgG2, IgG3, and IgG4. Multimers of IgG1 have also been described by the addition of IgM tailpiece and Cys309 in the IgG1 Fc as stated above (see IgG multimerization augments FcγR binding). IgG hexamers created using the IgM tailpiece strategy also showed improved binding to C1q and C5b relative to wildtype IgG1 (55). Thus, multimerization of IgG is another method in addition to point mutations and IgG1/IgG3 cross-isotype antibodies to increase C1q binding affinity.

#### Glycoengineering to Improve Complement Binding

The Asn297 glycan within the CH2 domain of the Fc can be modified to improve CDC activity (56, 57, 98–101). In a large screen of 20 different glycoforms of anti-trinitrophenyl hapten IgG1 Fc an overabundance of galactosylation increased C1q binding and CDC activity compared to the unmodified glycoform of IgG1 (57). Galactosylation appeared to be the principal glycan residue that affected CDC activity as significant positive correlations were observed between abundance of galactosylation on Fc and CDC potency (57). In a separate study Peschke et al. confirmed the importance of galactosylation for CDC activity using a different antibody specificity and multiple IgG subclasses. Galactosylation of the IgG1 Fc improved CDC activity of the anti-CD20 antibody Rituxumab against Raji B cells *in vitro* (56). The improved CDC activity conferred by galactosylation of the Fc was applicable to IgG3, but was not applicable to IgG2 or IgG4 in this *in vitro* model (56). The improved CDC activity conferred by galactosylated Fc was not due to changes in antigen binding, but instead was associated with enhanced C1q binding (56, 57). Overabundance of galactosylation on IgG1 Fc also improved thermostability when measured by differential scanning calorimetry (99). Thus, galactosylating the Fc is one strategy for producing a stable biologic with highly potent CDC activity.

## Improved Antibody Half-Life Circulation

In addition to improving antibody effector functions by increasing affinity for activating FcγR and C1q, Fc optimization efforts have also tried to improve antibody circulation *in vivo*. *In vivo* IgG catabolism is regulated by its interaction with the neonatal Fc receptor (FcRn) (102). The FcRn binds to IgG at the junction of the CH2 and CH3 domains in a pH dependent manner (102–104). IgG is endocytosed by cells where it can be shuttled to lysosomes or recycled back to the cell surface (105). Binding of IgG to FcRn at low pH (pH < 6.5) in the endosomes allows the antibody to be trafficked with the FcRn back to the cell surface (106, 107). Poor binding to FcRn at pH < 6.5 results in the antibody being trafficked to the lysosome and degraded (105). At the physiologic pH of the extracellular environment IgG has weak affinity for FcRn which results in its release from the FcRn back into circulation (105). The pH dependent binding is regulated by protonation of His310, 435, 436 in the Fc at low pH (108). The protonation creates positively charged residues that can bind to negatively charged Glu117, Glu132, and Asp137 in the FcRn (109).

### Point Mutations to Increase FcRn Binding Affinity

In a global approach to increasing IgG1 half-life, alanine scanning mutagenesis of the Fc was performed. In this screen 17 amino acids that affect IgG Fc binding to FcRn were identified (38). Among the 17 amino acids Asn434 and Glu380 showed large increases in affinity when mutated to alanine (38). The Asn434Ala mutation has been useful for countering the poor FcRn affinity that can result from the introduction of FcγR affinity-optimizing mutations (38), thus Asn434Ala is typically added to Ser298Ala, Glu333Ala, and Lys334Ala to create a AAAA variant with enhanced FcγR binding and normal or slightly improved FcRn binding (Table 2) (38). Additional half-life mutations were identified by sequentially performing random and directed evolution screens of phage libraries. The phage binding was done at pH 6 to mimic endosomal pH, and elution was done at pH 7.4 to find variants that did not bind at physiologic pH. Six collections of mutations were identified that improved FcRn binding across three different assays including Glu294deletion/Thr307Pro/Asn434Tyr (termed C6A-66) and Thr256Asn/Ala378Val/Ser383Asn/Asn434Tyr (referred to as C6A-78) (116). Asn434Tyr was among the most common mutations found in each collection (114). One of the differences between these collections of mutations was the ability to bind to FcγRIIIa, thus one could extend half-life while also retaining ADCC activity or knocking out ADCC activity (114). In more recent work, the C6A-66 collection of mutations were analyzed further since it showed only a moderate increase in FcRn binding, but had the best serum half-life *in vivo*. The collection of mutations were studied as individual mutations to elucidate the function of the deletion of Glu294 (115). This deletion resulted in higher sialylation of the Asn297 glycan on the Fc (115). The abundance of sialic acid was necessary for the increase in antibody half-life *in vivo* (115). Thus, increased FcRn binding was not the only factor that contributed to increased half-life. Sialylation also has a role in regulating serum half-life (115).

**Table 2:** Fc modifications to improve antibody circulation half-life.

**Modifications or mutations (reference)**	**Abbreviated name**	**Phenotype**	**Enhanced function**
Arg435His (110)	His435	• Increased binding to FcRn at low pH	Extended half-life
Asn434Ala (38)	A	• Increased binding to FcRn at pH6	Extended half-life
Met252Tyr/Ser254Thr/Thr256Glu (111)	YTE	• Slowed off-rate for Fc and FcRn • Increased FcRn affinity • Decreased ADCC	Extended half-life
Met428Leu/Asn434Ser (112)	LS	• Increased affinity to and slowed off-rate for FcRn at pH6 • No change in ADCC	Extended half-life
Thr252Leu/Thr253Ser/Thr254Phe (113)	LSF	• Increased binding to FcRn at pH < 6.5	Extended half-life
Glu294delta/Thr307Pro/Asn434Tyr (114)	C6A-66	• Increased binding to FcRn at pH < 6 • No binding to FcRn at pH7.4 • Decreased FcγRIIa binding and ADCC	Extended half-life
Thr256Asn/Ala378Va l/ Ser383Asn/Asn434Tyr (114)	C6A-78	• Increased binding to FcRn at pH < 6 • No binding to FcRn at pH7.4	Extended half-life
Glu294delta (114, 115)	Del	• Increased sialylation	Extended half-life

Ghetie et al. also created large libraries of random mutations of Thr252, Thr253, and Thr254 in the mouse Fc and screened them for binding to mouse FcRn using a bacteriophage display platform (113). The three sites were chosen based on their proximity to the FcRn binding site on Fc. From the phage library the collection of Thr252Leu, Thr253Ser, and Thr254Phe was identified that had significantly longer half-life in wildtype mice. These three mutations, termed LSF, did not affect association rates of Fc with FcRn but did slow the dissociation rate of Fc from FcRn at pH6 (113). This result indicated for mouse antibodies that that position 252, 254, and 256 could be manipulated to increase antibody half-life. Hence in a later study, phage display libraries of human IgG1 were used to identify analogous mutations at positions 252, 254, and 256 (111). In the human IgG1 Met252Tyr, Ser254Thr, and Thr256Glu was observed in a high abundance among the clones isolated from the phage library. Inclusion of these 3 mutations, often called YTE, in the IgG1 Fc resulted in a 10-fold slower dissociation rate of Fc and FcRn. Overall, the YTE mutations enhanced the apparent equilibrium rate constant 3-fold for Fc binding to FcRn. To determine whether the increased binding to FcRn *in vitro* translated to improved pharmacokinetics in primates, wildtype IgG1 or a YTE variant were infused into cynomolgus monkeys and serum half-life was compared. The YTE antibody possessed a 4-fold increase in serum half-life as compared to the wildtype antibody (64). Additionally, there was a higher concentration of infused IgG measured in bronchioalveolar lavage fluid when the Fc included the YTE mutations (64). The pharmacokinetic profile of an IgG1 possessing the YTE mutations was determined in a phase 1, double-blind, dose escalation study. In this study the YTE-variant of IgG1 had a serum half-life of 80–112 days (117). Based on the typical serum half-life of IgG1 being 21 days, the YTE mutation seems to increase half-life 4–5-fold in humans (117). In a second study YTE was introduced into motavizumab and their half-life was directly compared in humans. Consistent with the first study, serum half-life was extended 2–4-fold compared to wildtype IgG. Remarkably, the individuals that received the YTE variant had functional therapeutic antibody present in their serum 240 days after antibody infusion (118). Thus, the YTE mutations raise the possibility of long-acting antibody-based biologics that could suppress or protect from disease in humans. While YTE improves half-life of the antibody, it also eliminates ADCC activity of the antibody (64). This pitfall can be counteracted by the addition of ADCC-enhancing mutations, such as DLE (64). Therefore, YTE mutations should be used alone only when the antibody does not need to mediate ADCC.

Another set of mutations that improve antibody half-life was discovered by Zalevsky et al. using rational protein design. Introduction of Met428Leu and Asn434Ser mutations (referred to as the LS mutations) in IgG1 Fc resulted in a decrease in the dissociation rate and an 11-fold improvement in binding affinity between Fc and human FcRn at pH6 [Figure 2A; (112)]. In contrast to YTE, LS mutations did not significantly reduce ADCC activity (119). In cynomolgus macaques, the LS mutations conferred a 3-fold increase in antibody half-life (112). A similar increase of 3–4-fold in serum antibody half-life was seen in human FcRn transgenic mice (112). The improvement in function conferred by the LS mutations was tested by engrafting tumors into the human FcRn transgenic mice and infusing wildtype or LS-mutant IgG1 (112). For two different cancer immunotherapeutic antibodies the LS mutant IgG1 inhibited tumor growth significantly better than the wildtype IgG1 (112). Since the initial description of the LS mutations, multiple groups have shown these mutations boost antibody half-life in cynomolgus macaques (120, 121) and rhesus macaques (119, 122). The LS mutations have been helpful in sustaining protection against HIV-1 infection in animal models (119, 122, 123). The incorporation of LS resulted in increased antibody concentrations at mucosal sites and prolonged serum half-life (119, 122). Together these attributes resulted in improved protection afforded by optimized IgG1 in macaque models of HIV-1 infection (119, 122, 123). Clinical trials are planned to administer anti-HIV-1 IgG1 antibodies encoding the LS mutation. The extent to which the improved pharmacokinetic profile translates from macaques to humans will be determined, and whether longer half-life improves therapeutic efficacy will be evaluated. Novel mutations to improve antibody half-life are still being pursued. Approaches to improve upon the LS mutations include finding mutations that completely eliminate Fc binding to FcRn at physiologic pH, while also enhancing binding at low pH.

### Cross-Subclass Point Mutations to Enhance FcRn Binding

IgG1 makes these 3 productive Fc:FcRn contacts and has a half-life of 21 days (109). In contrast to IgG1, IgG3 alleles typically encode arginine at position 435 instead of histidine. IGHG3^*^17, IGHG3^*^18, and IGHG3^*^19 alleles are the exceptions, which encode histidine like IgG1. The presence of arginine vs. histidine confers a serum half-life of only 7 days (124). *In vitro* competition assays suggest that IgG1 with His435 outcompetes IgG3 with Arg435 for FcRn binding (110). Furthermore, Arg435 seems to increase binding of IgG3 to FcRn at physiologic pH (110), which could result in more IgG3 being absorbed to epithelial cells expressing FcRn hindering the ability of IgG3 to freely circulate in serum. To increase IgG3 half-life, position 435 was changed to histidine, which boosted FcRn binding at low pH (110). Consistent with the increased binding, serum concentrations of IgG3 are higher in individuals who express an allelic variant of IgG3 encoding His435 and infusion of IgG3 encoding His435 is more efficient (110, 125). Thus, modulation of the pH sensing ability of IgG3 Fc is one mechanism for boosting its serum half-life.

## Antibody Fc Engineering for the Ablation of Effector Functions

While Fc optimization has focused heavily on gain-of-function modifications, in certain situations it can be beneficial to eliminate antibody Fc function. These situations include antibodies that are used as **(1)** receptor agonists to crosslink receptors and induce signaling, **(2)** receptor antagonists to block receptor:ligand interactions to prevent signaling, or **(3)** drug delivery vehicles to deliver drug to antigen-expressing target cells (Figures 1C,D). In these instances Fc engagement of receptors on effector cells or engagement of C1q is not wanted, because it can lead to undesired killing of biologically-important cells expressing the receptor or recruitment of drug-conjugated antibodies to off-target cells (126, 127). Below, strategies to eliminate FcγR binding and complement protein C1q binding are described.

### Ablation of FcγR Binding

#### Point Mutations to Ablate FcγR Binding

**Figure 3:**
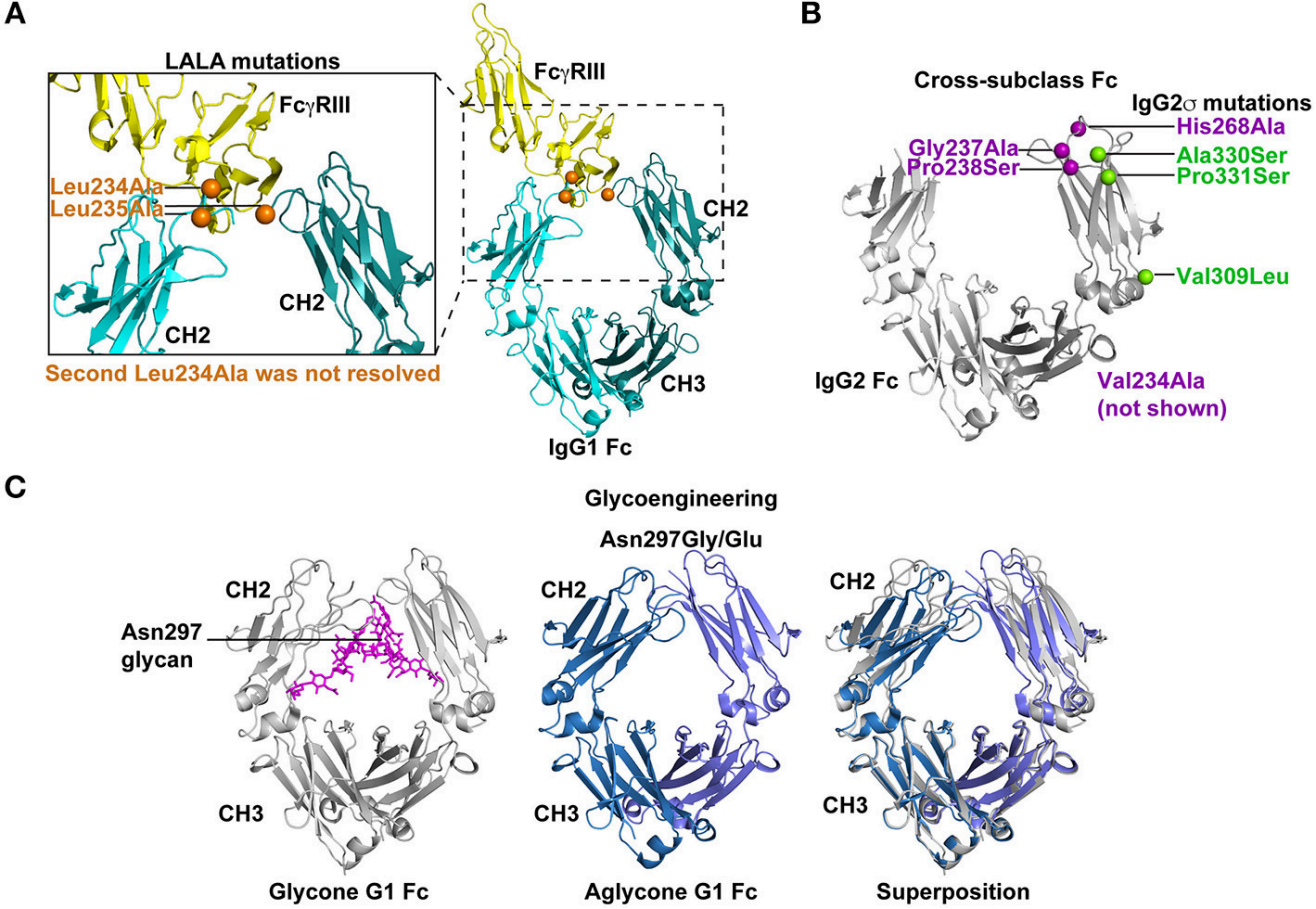
Strategies for silencing antibody effector functions. **(A)** Point mutations in the Fc have been identified that disrupt antibody effector functions. The elucidation of key amino acids in the interaction of Fc with FcγRs has led to collections of point mutations that can eliminate or drastically reduce Fc binding to specific FcγRs. The Leu234Ala/Leu235Ala (LALA) mutations are perhaps the most commonly used mutations for disrupting antibody effector function (130, 131). As shown in the co-crystal structure (PDB: 1T83) with orange spheres the LALA mutations are proximal to FcγRIII (yellow) when it binds the IgG1 Fc (light and dark teal). These mutations can be combined with other effector function silencing strategies to engineer a Fc that is devoid of any FcγR binding or C1q binding. Leu 234 was only resolved in one of the chains of the Fc region. **(B)** Effector functions can be disrupted by exchanging amino acids between two Fc molecules from different IgG subclasses. These cross-subclass Fc designs rationally combine mutations that knockdown binding to a given FcγR or complement protein. IgG2m4 and IgG2σ are two examples of engineered Fc regions that were generated by this approach (143, 144). IgG2σ is perhaps the most effector function silent Fc and it combines cross-subclass mutations Val309Leu, Pro331Ser, and Ala330Ser (green spheres) with four additional mutations not naturally found in human Fc sequences (purple spheres) (144). The crystal structure of the IgG2 Fc (gray) encoding these mutations (green and blue spheres) showed the CH2 domains moved farther apart from each other. Also, Asp270 and Pro329, which are essential for binding to FcγR and C1q, were repositioned (PDB:4L4J; 145). Position 234 was not visible in the crystal structure and is not shown in figure. **(C)** Removal of the Asn297 glycan in the IgG Fc severely reduces Fc binding to FcγRs by inducing a Fc closed conformation. Several Fc designs have removed the N-linked glycosylation site at position 297 by introducing Asn297Gly or Asn297Glu changes (151, 152). The crystal structure of glycosylated IgG1 Fc (gray) is shown with the N297 glycan (magenta; PDB:4BYH). The introduction of a Gly or Glu residue at position 297 produces an aglycone IgG1 Fc (blue and lilac; PDB:3S7G). Superposition of the glycone and aglycone Fc crystal structures shows the lilac and blue CH2 domains in the aglycone are closer in proximity than the gray CH2 domains. The altered CH2 conformation has been hypothesized to be the structural explanation for reduced FcγR binding by the aglycone Fc.

One of the earliest antibodies used in humans was OKT3 to prevent transplant rejection (1). Despite humanization of the antibody, this antibody induced proinflammatory cytokine secretion, which resulted in toxicity (128, 129). The cytokine secretion was due to binding of OKT3 to CD3 followed by crosslinking of FcγRs on T cells (129). To alleviate the cytokine induction from T cells the Fc region of the antibody was mutated to eliminate FcγR binding (129). A single mutation of Leu235Glu was sufficient for knocking out binding to Fc receptors on U937 cells (129). Furthermore, the 100-fold reduction in binding to FcγR also resulted in lower T cell activation and proliferation in the presence of the Leu235Glu Fc mutant IgG1. Building upon this initial mutation it was found that the combination of Leu234Ala and Leu235Ala (commonly called LALA mutations) eliminated FcγRIIa binding [Table 3 and Figure 3A; (130, 131)]. These two mutations were later shown to eliminate detectable binding to FcγRI, IIa, and IIIa for both IgG1 and IgG4 (153). The use of LALA appears to be more effective than either Leu234Ala or Leu235Ala alone. Some groups have seen that the Leu235Glu mutation knocks out FcγRI binding (129). However, there are reports that this high affinity receptor still binds to IgG1 Fc when this mutation is present (132). Similarly, Leu234Ala single mutant Fc still possessed detectable binding to the high affinity Fc receptor FcγRI at least in some assays (131). Both the single and double mutations at position 234 and 235 reduced ADCC activity mediated by PBMCs and nearly ablated ADCC mediated by monocytes (132). Nonetheless, the LALA mutations have been tested in humans in a phase I clinical trial. Anti-CD4 antibody OKT3 encoding the LALA mutations was administered for the treatment of acute renal allograft rejection. The IgG1 with LALA mutations caused minimal adverse reactions and was able to reverse allograft rejection in 6 of 7 individuals (154). One common pairing, in addition to the LALA pairing, is Ser228Pro paired with Leu235Glu, which has been called the SPLE or PE mutations (133). The SPLE mutations have been introduced into IgG4, which has low binding to FcγR initially perhaps due to the Phe234 residue that differs from Leu234 found in IgG1 (155). This combination dramatically reduced FcγRI binding to IgG Fc to barely detectable levels by surface plasmon resonance (133), without reducing circulating half-life in rats (155). The effect of the Ser228Pro is thought to be mostly to improve stability of IgG4 (155).

**Table 3:** Fc modifications to silence antibody effector function.

**Modifications or mutations (reference)**	**Abbreviated name**	**Phenotype**	**Reduced effector function**
Leu235Glu (129)	LE	• Decreased binding to cell surface FcγRs	ADCC
Leu234Ala/Leu235Ala (130–132)	LALA	• Decreased binding to FcγRI, II, III	ADCC ADCP CDC
Ser228Pro/Leu235Glu (133)	SPLE in IgG4	• Decreased FcγRI binding • Half-life was unchanged	
Leu234Ala/Leu235Ala/Pro329Gly (134)	LALA-PG	• Eliminated binding to FcγRI, II, III, C1q	ADCP
Pro331Ser/Leu234Glu/Leu235Phe (135, 136)	TM	• Decreased binding to FcγRI, II, III and C1q	CDC
Asp265Ala (134, 137)	DA	• Decreased binding to FcγRI, II, III	ADCC ADCP
Gly237Ala (138)		• Decreased binding to FcγRII	ADCP
Glu318Ala (138)		• Decreased binding to FcγRII	ADCP
Glu233Pro (38)		• Decreased binding to FcγRI, II, and III	
Gly236Arg/Leu328Arg (139, 140)	GRLR	• Decreased binding to all FcγR	ADCC
IgG2-IgG4 cross-subclass (141, 142)	IgG2/G4	• Decreased binding to FcγRs and C1q	
His268Gln/Val309Leu/Ala330Ser/Pro331Ser (143, 144)	IgG2m4	• Decreased binding to all FcγR • Decreased C1q binding	ADCC ADCP CDC
Val234Ala/Gly237Ala/Pro238Ser/His268Ala/Val309Leu/Ala330Ser/Pro331Ser (144)	IgG2σ	• Near complete elimination of FcγRI, IIa, IIb, and IIIa binding • Decreased C1q binding • Binds FcRn	ADCC ADCP CDC
Leu234Ala/L235Ala/Gly237Ala/P238Ser/His268Ala/Ala330Ser/Pro331Ser (144–146)	IgG1σ	• Near complete elimination of FcγRI, IIa, IIb, and IIIa binding • Binds FcRn	ADCC CDC
Ala330Leu (89)	AL	• Decreased C1q binding • Part of DLE mutations	CDC
Asp270Ala (89)		• Decreased C1q binding	CDC
Lys322Ala (89)		• Decreased C1q binding	CDC
Pro329Ala (89)		• Decreased C1q binding	CDC
Pro331Ala (89)		• Decreased C1q binding	CDC
IgG2-IgG3 cross-subclass (51)		• Decreased C1q binding	CDC
High mannose glycosylation (147, 148)		• Decreased C1q binding	CDC
Val264Ala (137)		• Decreased C1q binding	CDC
Phe241Ala (137)		• Decreased C1q binding	CDC
Asn297Ala or Gly or Gln (32, 149–152)		• Decreased binding to FcγRI and IIIa • Decreased C1q binding	ADCC ADCP CDC
S228P/Phe234Ala/Leu235Ala (144)	IgG4 PAA	• Decreased binding to FcγRI, IIa and IIIa	ADCC CDC

The LALA mutations have provided a foundation for the addition of other mutations or new modifications to Leu235. Building upon the LALA mutant phenotype, Oganesyan et al. mutated Leu234 and Leu235, but also added Pro331Ser to the Fc design to completely abrogate binding between Fc and FcγRs (135). The triple mutant Pro331Ser, Leu234Glu, and Leu235Phe eliminated all FcγR binding (135) without disrupting the overall conformation of the Fc (135). Similarly, Pro329Gly mutation was added to the LALA mutations, which inhibited binding to murine FcγRI, II, and III by IgG2a Fc (134, 156). The amino acid at 329 was changed, because this residue makes contact with Trp108 and Trp131 of FcγRIIIa (61). The LALA-PG was an improvement over LALA mutations alone in that they nullified Fc function in mouse and human IgG (134), whereas LALA alone still retains murine FcγRIII binding to murine IgG2a (157). The significance of the LALA-PG mutations are that observed results in murine models are expected to more accurately translate to humans since the mutations confer a similar phenotype for both murine IgG2a and human IgG1 (134).

The LALA mutations are among the most common point mutations used to disrupt Fc receptor binding, however other sites have been reported to knockout Fc receptor binding. Using a panel of 32 site-directed alanine mutations in IgG Fc Lund and colleagues showed that Gly237 and Glu318 were required for FcγRII binding (131, 138, 158). This lack of binding resulted in poor phagocytosis *in vitro* (138). Additional alanine scanning mutagenesis experiments determined 9 different amino acid substitutions that resulted in loss of binding to FcγRI, IIa, IIb, and IIIa. Notably, Asp265Ala and Glu233Pro mutations reduced binding to all 4 receptors by >80% (38). Ala318, Ala237, Ala265, and Pro233 represent a collection of mutations that can be used in various combinations to eliminate Fc receptor binding to Fc (134, 137, 138).

#### Cross-Subclass Fc Designs Eliminate FcγR Binding

To silence the effector functions of Fc, large portions of Fc regions from different subclasses have been exchanged to generate cross-subclass Fc regions (141). These designs aim to silence the Fc effector functions by combining CH domains from different subclasses that lack opposing functions. For example, IgG2 has poor FcγR binding but binds C1q, and IgG4 lacks C1q binding but reacts with FcγRs (142). Hence, combinations of IgG2 and IgG4 CH domains have been constructed that are devoid of both C1q and FcγR binding (141, 142). Typically, in the IgG2/G4 chimeras the hinge and CH1 domain originates from IgG2 and the CH2 and CH3 domains are from IgG4 (141, 142).

Using a different approach to the same concept An and colleagues compared the amino acid sequences of different IgG subclasses and introduced mutations into IgG2 that would completely eliminate FcγR binding. The aim of this approach is to introduce natural amino acids into the Fc so that the Fc would not be immunogenic. The investigators made conservative changes in the IgG2 primary sequence resulting in His268Gln/Val309Leu/Ala330Ser/Pro331Ser mutations (143). This cross-subclass design was termed IgG2m4 and lacked binding to all FcγR (143). The circulating half-life of this antibody was comparable to wildtype IgG in macaques, which suggested the transplantation of IgG4 residues did not make the IgG2 more immunogenic (143).

More recently, Vafa et al. combined many of the mutations that have been discovered over the last 25 years to create an engineered construct called G2σ (Figure 3B). This construct included Val234Ala/Gly237Ala/Pro238Ser/His268Ala/Val309Leu/Ala330Ser/Pro331Ser mutations where many of the mutations were previously established as silencing mutations and the remaining mutations were selected as cross-subclass mutations that introduced IgG4 residues into IgG2 (144). In direct comparisons with IgG1, IgG2, IgG4, and IgG2m4, IgG2σ had the most profound elimination of binding to FcγRI, IIa, and IIIa (144). However, both IgG2m4 and IgG2σ lacked *in vitro* ADCC activity mediated by human PBMCs effector cells and possessed very little ADCP activity against breast cancer cell lines (144). Given the success of IgG2σ at ablating Fc effector functions, the design was translated to IgG1 and IgG4 (145, 146, 159). The IgG1σ (Leu234Ala/L235Ala/Gly237Ala/P238Ser/His268Ala/Ala330Ser/Pro331Ser), IgG2σ, and an IgG4 Fc encoding S228P/Phe234Ala/Leu235Ala mutations (termed IgG4 PAA) versions of Fc were compared for binding to FcγRs from multiple species (144, 145). IgG1σ and IgG2σ lacked binding to FcγRI and III (145). For FcγRIIa and IIb extremely weak binding could be seen to IgG1σ and IgG2σ at high concentrations of antibody (145). Both IgG1σ and IgG2σ exhibited lower binding to FcγRs than IgG4 PAA (145). IgG4 PAA also showed species-specific differences in binding to FcγRs, whereas IgG1σ and IgG2σ lacked binding for human, macaque, and mouse FcγRs. Therefore, IgG1σ and IgG2σ are among the most effective mutations for knocking out Fc effector function.

Mimoto et al. sought to use FcγRIIb as a way to capture immune complexes on the surface of FcγRIIb-expressing B cells (160). Thus, they engineered the Fc to selectively bind to FcγRIIb with a 200-fold increase in affinity, and a 10-fold lower affinity for the other FcγRs (160). The improved affinity for FcγRIIb conferred the desired boost in B cell presentation of peptides to T cells *in vitro* (160). Since FcγRIIb is an inhibitory receptor these mutations could be used to silence effector function by changing the ratio of Fc binding to activating vs. inhibitory receptors.

### Ablation of C1q Binding to Reduce Complement Dependent Cytotoxicity (CDC)

#### Point Mutations to Ablate Complement Binding

Inducing the complement cascade has been associated with antibody injection site adverse reactions (161, 162). Therefore, eliminating C1q binding to Fc—the initial event in the activation of antibody-dependent complement cytotoxicity (163)—has been a goal of Fc optimization. One of the benefits of the mutations engineered to eliminate FcγR binding is that many of them eliminate C1q binding too. In a structure-guided screen of Fc mutations, an Ala330Leu mutation was observed to decrease C1q binding (39). As stated above this mutation also eliminated FcγRIIb binding (39). In *in vitro* assays the Ala330Leu mutation reduced the ability of the antibody to mediate complement-dependent cytotoxicity (CDC) of target cells, presumably because Ala330Leu disrupted C1q binding to Fc. However, not all amino acids introduced at position 330 disrupted C1q binding, thus the effect was specific to the introduction of only certain amino acids at position 330 (39). It should be noted that Ala330Leu is one of the mutations in the set of mutations comprising the DLE mutations (Ser239Asp Ile332Glu Ala330Leu) that improve FcγR-mediated effector functions. The DLE optimization eliminates CDC activity mediated by the antibody, which can be rescued by removing the Ala330Leu mutation from the set. In instances where CDC causes adverse reactions to antibody administration the DLE mutations may be able to enhance FcγR-mediated effector functions and eliminate injection site reactions (39).

Additional amino acids that reduce C1q binding were identified by an alanine scan of the Fc. Asp270, Lys322, Pro329, and Pro331 were all implicated as sites in IgG that confer binding to C1q (89). Among these amino acid positions Asp270Ala and Pro329Ala, showed the most pronounced deficiency in complement activation and C1q binding across multiple concentrations of serum C1q (89). The Leu234Glu/Leu235Phe/Pro331Ser triple mutant Fc lacks binding to FcγRs, but also these mutations eliminate Fc binding to C1q (135). Similarly, the creation of IgG2m4 not only eliminates FcγR binding but also eliminates C1q binding (143). Vafa et al. examined the CDC activity of IgG2m4 (His268Gln/Val309Leu/Ala330Ser/Pro331Ser) and IgG2σ (Val234Ala/Gly237Ala/Pro238Ser/His268Ala/Val309Leu/Ala330Ser/Pro331Ser) formats of Rituxan (144). Neither antibody format conferred CDC against a lymphoma cell line using human serum complement (144) showing both are potential designs for eliminating complement-mediated functions. The structure of IgG2σ Fc was solved to 1.9 angstroms and showed that it is in a more open conformation meaning the CH2 domains of the Fc are spaced relatively far apart (144). Moreover, the loop containing Leu328 is repositioned compared to wildtype IgG2 Fc. Thus, it is postulated that the change in conformation results in reorientation of Asp270 and Pro329, which eliminates FcγR and C1q binding to IgG2σ (144).

#### Cross-Subclass Fc Regions to Ablate Complement Activation

Fundamental knowledge of how each IgG subclass interacts with complement allows for fine tuning of CDC. For example, IgG2 can have moderate to low CDC activity (51). Thus to reduce IgG3 CDC activity the CH2 domain of IgG2 can be used to replace the CH2 domain of IgG3 (51). Similarly, to ablate IgG1 CDC activity a Pro331Ser mutation was introduced based on the fact that IgG4 has Ser331 and lacks CDC activity (136). These mutations are an example of how basic science can be applied to the design of antibody-based biologics. Also many of the point mutations made to knockout C1q binding are cross-subclass mutations (Table 3).

#### Glycoengineering to Ablate FcγR and C1q Binding

The Fc of IgG contains an N-linked glycosylation site at position 297 [Figure 3C; (72)]. Typically the glycan present at N297 is a complex biantennary glycan (164, 165). The modification of this glycan to high mannose glycan reduced the affinity of IgG1 Fc for C1q, which in turn reduced CDC activity (147, 148). Inhibiting the incorporation of galactose or sialic acid into carbohydrate synthesis did not dramatically silence immune effector functions of the antibody Fc (147). While glycans devoid of galactose or sialic acid appear to function normally, mutations in the Fc that knockout C1q and FcγRI binding can also lead to an increase in galactosylation and sialylation of the Asn297 glycan eliminate (137). Galactosylation and sialylation-increasing mutations include Phe241Ala, Val264Ala, and Asp265Ala mutations (137). Whether the change in glycosylation profile has a role in reducing C1q binding or is an unrelated bystander effect is not clear. In one study, sialylation of the Fc reduced C1q binding 4-fold suggesting hypersialylation could directly impair CDC responses (101). However, hypersialylation reduces terminal galactosylation complicating which factor contributes to the reduced C1q binding (101).

Another common method to eliminate Fc effector function has been to completely remove the glycosylation site by substituting alanine, glutamine, or glycine at position 297 (32, 149–152). The removal of the glycosylation site dramatically reduced IgG1 binding to FcγRI and C1q [Figure 3C; (137, 152, 166)]. In the context of IgG3, the Fc lacking glycosylation—the aglycone Fc—has reduced binding to FcγRI and C1q. *In vitro*, the aglycone IgG3 Fc loses the ability to mediate ADCC via FcγRIIIa (151, 152). However, removal of Asn297 glycan reduces, but does not eliminate, binding to mouse Fc receptors (157). Additionally, it is thought that avidity can overcome the low binding affinity to FcγRI conferred by mutating Asn297 (144). Therefore, on monocytes and macrophages where FcγRI is expressed at a high density the Asn297Gly mutation may not be sufficient to eliminate all binding. Lo et al. combined the Asn297Gly mutation with Asp265Ala mutation to further reduce FcγR binding to Fc. As stated above each of these mutations reduces FcγR and C1q binding individually and when combined showed a further reduction in Fc binding to FcγRs and C1q (134). Combinations such as the Asn297Gly/Asp265Ala are useful for near complete knockout of binding between Fc and FcγRs or C1q.

The mechanism behind the aglycone reducing Fc binding to C1q and FcγRs is not fully understood. The aglycone Fc is more susceptible to protease cleavage, which suggests the structure of the glycone Fc differs from that of the aglycone (152). Nuclear magnetic resonance studies have similarly suggested structural perturbations are present in the aglycone Fc (32, 73, 151). The clearest evidence for structural changes in the agylcosylated Fc was provided by the crystal structure of mouse Fc without the N297 glycan (167). The CH3 domains appeared identical, but the CH2 domains of aglycone Fc and glycosylated Fc differed in position by 10 to 14 angstroms (167). In the aglycone Fc the CH2 domains were closer together than in the glycosylated Fc, thus the aglycone had a “closed” conformation. This closed conformation was not unique to mouse Fc. Crystal structures of both aglycosylated human IgG1 Fc (168) and IgG4 Fc (169) have shown the CH2 domains of a single Fc to undergo rigid-body movements of 10–20 angstroms to be closer in proximity to each other when aglycosylated (Figure 3C). The closed conformation of the aglycone Fc is mediated, at least in part, by the perturbed C'E loop (149, 150, 167, 168, 170). In total, eliminating Fc glycosylation induces a closed conformation that confers silencing of the Fc effector functions.

## Conclusions

Many approaches including phage display, alanine scanning mutations, and structure-based design have all been successful in optimizing the Fc of antibody-based biologics (38, 39, 113). Underlying the optimization of the Fc is modulating its ability to bind to Fc receptors, C1q, and FcRn. These interactions can be modulated by the introduction of point mutations, inserting or deleting amino acids, modifying glycan composition, or appending protein domains (171). Overall, strengthening or disrupting Fc interactions with its binding partners as measured by *in vitro* affinity has translated to the desired outcome *in vivo*. The optimization of Fc for specific functions not only can improve *in vivo* functions, but it also provides a means to dissect the importance of specific Fc receptors (172) and downstream CDC, ADCC, phagocytosis, or circulation half-life in treating specific diseases (119, 173). While antibody-based biologics have been successful in the treatment of disease, new opportunities exist for antibody biologics as durable prevention strategies for infectious diseases (174–176). The antibody-mediated prevention (AMP) study will test the efficacy of monoclonal antibody passive infusion to prevent HIV-1 infection in 2,700 participants (NCT02716675). This phase 2b trial could provide the first proof-of-concept that neutralizing antibodies can provide protection from HIV-1 infection in humans. The VRC01 antibody that will be used contains a wildtype Fc; however, the next generation of HIV-1 antibody protection studies will likely include combinations of Fc-optimized HIV-1 neutralizing antibodies with prolonged circulation half-life (116), since longer half-life was important for protection in preclinical nonhuman primate studies (119, 122, 123). The ability to optimize the Fc region of antibodies continues to be a powerful approach for combating heritable diseases, infectious diseases, and cancer.

## Author Contributions

The author confirms being the sole contributor of this work and has approved it for publication.

### Conflict of Interest Statement

The author declares that the research was conducted in the absence of any commercial or financial relationships that could be construed as a potential conflict of interest.
